# *SOCS1* insufficiency in systemic lupus erythematosus in a child: a case report

**DOI:** 10.3389/fped.2025.1618483

**Published:** 2025-07-18

**Authors:** Lu Cao, Qin Wang, Huating Zhang, Jianjiang Zhang

**Affiliations:** Department of Pediatrics, The First Affiliated Hospital of Zhengzhou University, Zhengzhou, Henan, China

**Keywords:** systemic lupus erythematosus, *SOCS1* insufficiency, child, phospholipid antibody, case report

## Abstract

This case report details a 6-year-old Han Chinese girl diagnosed with Systemic Lupus Erythematosus (SLE) associated with a frameshift variant in the *SOCS1* gene. Initially presenting with fever and rash, the patient exhibited abnormal liver function, hypocomplementemia, and positive antinuclear and anti-dsDNA antibodies. Genetic testing identified a heterozygous frameshift mutation in the *SOCS1* gene, inherited from her mother. The girl was treated with intravenous methylprednisolone, oral prednisolone, hydroxychloroquine, and mycophenolate mofetil, leading to significant clinical improvement. Considering the clinically relevant variant in *SOCS1*, the findings suggest that identifying pathogenic genes can facilitate the development of new therapeutic targets and biomarkers, with JAK inhibitors showing promise for treating *SOCS1*-related conditions.

## Introduction

Systemic lupus erythematosus (SLE) is a chronic inflammatory condition caused by issues in immune regulation genes. Various inflammatory cytokines and genes contribute to the pathogenesis of SLE. The discovery of the Suppressor of Cytokine Signaling 1 (*SOCS1*) represented a significant advancement in understanding the regulation of the Janus kinase/Signal Transducer and Activator of Transcription (JAK/STAT) signaling pathways (1). *SOCS1* modulates cytokine signaling pathways and immune responses, including interferon-*γ* signaling, CD4T cell differentiation, influence on CD4/CD8T cell development and activation, and innate immunity (1). *SOCS1* insufficiency disrupts immune homeostasis through multiple mechanisms: it impairs regulatory T cell (Treg) function, promotes hyperactivation of CD8+ T cells with a memory-like phenotype, and enhances B cell autoantibody production via elevated B-cell activating factor (BAFF) secretion (2). These abnormalities are linked to uncontrolled STAT1/STAT5 phosphorylation in response to IFN-γ and IL-2, leading to a “cytokine hypersensitivity” state.

In murine models, *SOCS1* insufficiency or complete loss-of-function mutations induce lupus-like phenotypes, including autoantibody production, glomerulonephritis, and lymphoproliferation. For example, Fujimoto et al. (3) demonstrated that *SOCS1*+/− mice develop spontaneous autoimmunity with age, characterized by anti-dsDNA antibodies, immune complex deposition in the kidneys, and Th1/Th17 cell skewing, mirroring human SLE pathogenesis. Similarly, Hadjadj et al. (2) reported that *SOCS1* insufficiency in humans leads to early-onset autoimmunity, with two patients meeting SLE criteria and exhibiting mesangial proliferative glomerulonephritis and dysregulated B cell subsets.

The JAK/STAT pathway is crucial in various physiological and pathological processes, such as immune responses, cell proliferation, differentiation, and apoptosis.

This case underscores the importance of genetic analysis in pediatric SLE, especially in young patients with severe disease, a family history of relevant diseases, or an atypical response to treatment. Such analysis can be more targeted and practical, guiding accurate diagnosis and management. Whole Exome Sequencing (WES) shows potential in this regard. The potential link between SLE and *SOCS1* insufficiency lies in the latter's role in immune regulation. Further research is crucial to explore this relationship and its implications for treatment (2). In this study, we report a case of SLE in a child with a frameshift mutation in the *SOCS1* gene.

## Case presentation

**Figure 1:**
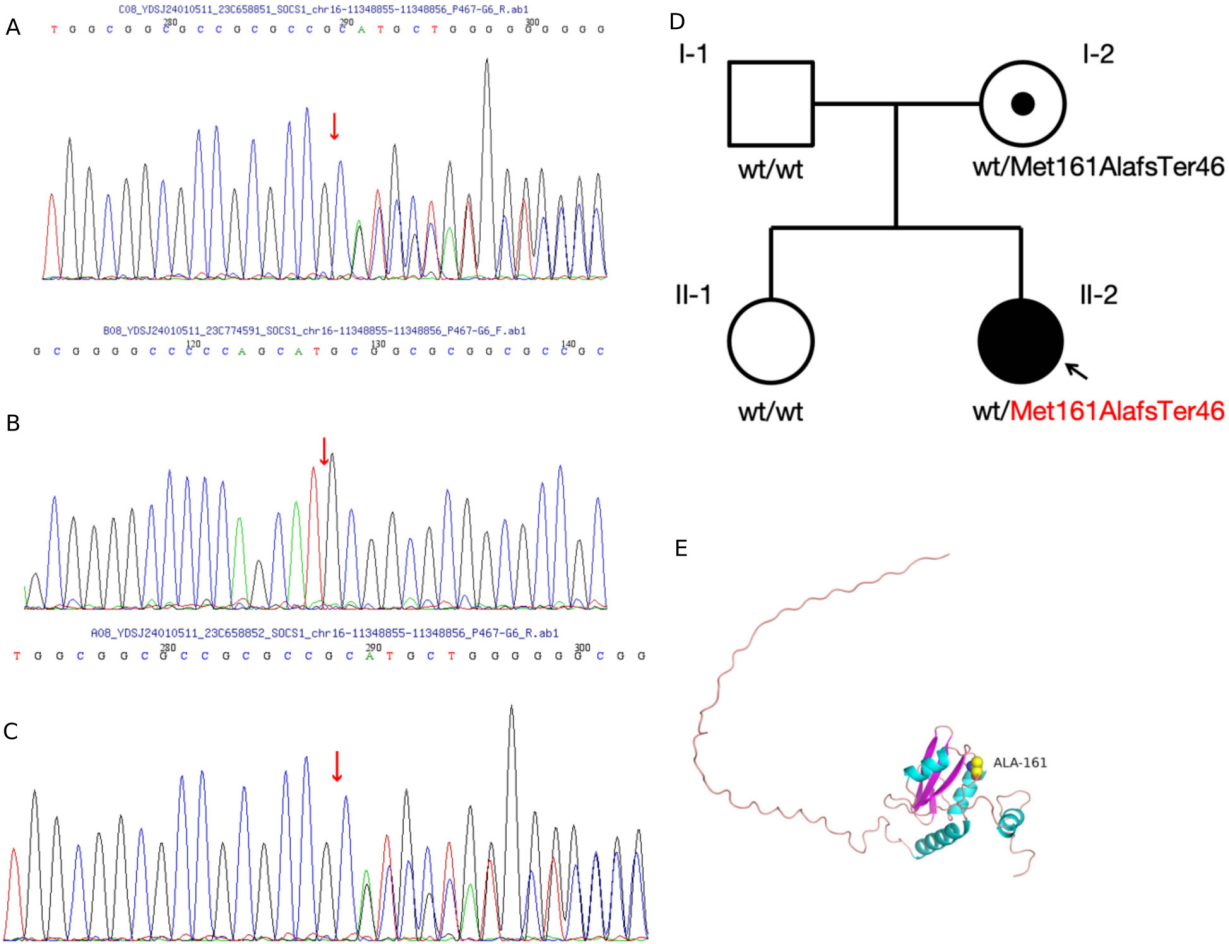
**(A–C)** sanger sequencing of identified alterations using whole exome sequencing. **(D)** Pedigree and variant of the *SOCS1* gene in the family. **(E)** Amino acid change from methionine to alanine at position 161, with an extension of 44 additional amino acids before termination of expression, resulting in the loss of 6 amino acids. This alteration affects the protein conformation. The predicted structure of *SOCS1*, modelled using SWISS-MODEL, is shown. Structural components are highlighted as follows: alpha helices (blue), beta sheets (purple), and loop structures (pink). Hydrogen bonds are represented by stick structures, with atoms coloured as follows: yellow for carbon, grey for hydrogen, blue for nitrogen, red for oxygen, and orange for sulfur.

At the onset of the illness, a 6-year-old girl presented with episodic fever and rash and was admitted to a local hospital. Laboratory analysis revealed a haemoglobin level of 96 g/L, a white blood cell count of 3.42 × 10^9^/L, an Epstein–Barr (EB) virus DNA quantification of 1.56E + 4 IU/ml, alanine aminotransferase (ALT) levels of 89 U/L, aspartate aminotransferase (AST) levels of 92 U/L, and 1 + urine protein. An upper abdominal computed tomography (CT) scan showed liver and spleen enlargement, pleural effusion on both sides, and inflammation of the lower lobe of the left lung. She was treated for infectious mononucleosis, and her temperature returned to normal, transaminases normalised, and the rash disappeared. A routine blood check-up at the local hospital revealed a white blood cell count of 3.27 × 10^9^/L, haemoglobin of 92 g/L, erythrocyte sedimentation rate (ESR) of 97 mm/h, ALT of 262 U/L, AST of 301 U/L, and proteinuria 1+. Upon referral to our hospital due to elevated transaminase levels, the patient presented with normal results for cytomegalovirus DNA and EB virus DNA tests. Further investigation revealed hypocomplementemia, prolonged coagulation screening with an activated partial thromboplastin time (APTT) of 69 s and a prothrombin time (PT) of 18.9 s, as well as proteinuria 1+ and microscopic haematuria of 11/μl. Antiphospholipid antibodies, anti-beta-2-glycoprotein-1 antibodies, and lupus anticoagulant were positive. Coagulation factors revealed mild decreases in the activities of Factors IX, XI, and XII. Laboratory tests showed positive antinuclear antibody (1/320 titre) and positive anti-double-stranded DNA (ds-DNA) [ds-DNA: 429 IU/ml]. A bone marrow biopsy showed no concern for malignancy or dysplasia. A lymph node biopsy showed reactive lymphoid hyperplasia. Magnetic resonance imaging (MRI) of the brain revealed no abnormality. The girl's past medical and family history did not include inflammatory or autoimmune diseases. A 24-h urine protein test showed 0.3 g. Based on her history and laboratory findings, the girl was diagnosed with systemic lupus erythematosus (SLE), meeting the 2019 EULAR/ACR classification criteria (4). The girl received intravenous methylprednisolone infusion (1.6 mg/kg/day, Max dose 48 mg/day) which was subsequently changed to oral prednisolone (2 mg/kg/day). Calcium supplementation, oral hydroxychloroquine (4 mg/kg/day), and mycophenolate mofetil (30 mg/kg/day) were also prescribed, along with liver protection and plasma transfusion. Symptomatic treatment included plasma transfusion and nutritional liver support. Oral administration of Benazepril tablets at 5 mg per day for 3 consecutive months to reduce urinary protein. Given the patient's initial symptoms consistent with infectious mononucleosis, which later progressed to manifestations suggestive of systemic lupus erythematosus, and considering the child's young age of six, a comprehensive whole exome sequencing (WES) analysis was conducted to identify potential pathogenic genetic variants for targeted treatment options. WES identified a frameshift variant of the *SOCS1* gene (exon 2, NM_003745.1: c.476_480dup; p.Met161AlafsTer46) inherited from her mother, who had no clinically significant systemic history (Figure 1). This variant has been previously reported in family C of a prior study (2), where a similar frameshift mutation in the *SOCS1* gene was associated with early-onset autoimmunity, including Evans syndrome and lymphoproliferative manifestations. The mutation introduces a premature stop codon and a 46-residue neopeptide within the *SOCS* box domain, predicted to disrupt JAK-STAT pathway regulation and cause loss of function. While the mother's carrier status does not negate the pathogenic role of the SOCS1 variant, the patient's severe, early-onset phenotype—combined with the variant's presence in a known disease-associated locus (2)—strongly supports its causal involvement. The absence of other identified pathogenic variants in SLE-associated genes (e.g., TNFAIP3, TREX1) further reinforces SOCS1 insufficiency as the primary driver.

After two months of treatment, the patient's liver function returned to normal. Significant clinical improvement was noted during hospitalisation and at the 6-month follow-up (Table 1).

**Table 1:** Details of the patient's clinical dates.

Admission time	2023.12	2024.01	2024.02	2024.03	2024.05	2024.07
Age	6y2m	6y3m	6y4m	6y6m	6y7m	6y9m
WBC,10^9^/L	4.38	16.67	9.75	8.22	7.32	7.61
HB, g/L	85	119	131	122	125	119
PLT, 10^9^/L	182	475	357	355	337	265
APTT, s (26–40)	54.8	45.6	30.7	48.9	44.3	44.9
PT, s (8.8–13.6)	18.9	10.7	10.10	11.5	10.9	12.1
Albumin, g/L	31.9	45.8	43.5	43.7	43.3	40.2
24 h urine protein, g	0.3	0.29	0.09	0.15	0.06	0.05
ANAs	1:320	1:1,000	1:320	1:320	1:320	1:100
anti-dsDNA, IU/ml (0–100)	543.5	251.8	360.9	242.2	275.4	218.9
IgG, g/L (5.66–14.25)	16.5	15.63	10.6	8.78	9.30	9.53
C3, g/L (0.91–1.57)	0.4	0.73	1.09	0.99	1.43	0.86
C4, g/L (0.14–0.44)	0.02	0.03	0.07	0.09	0.16	0.07
aCL-IgG, CU (0–20)	43	34.3	18.7	15.6	16.2	NA
aB2IgG, CU (0–20)	109.9	76.3	42.1	34.5	32.2	NA
CD19 + B cells (μl)	223	514	NA	NA	NA	NA
CD3 + CD4 + T cells (μl)	629	578	NA	911	NA	NA
CD3 + CD8 + Tcells count (μl)	942	975	NA	1,085	NA	NA
CD4 + T:CD8 + T ratio	0.66	0.59	NA	0.85	NA	NA

## Discussion

In this case report, a 6-year-old girl initially presented with symptoms suggestive of infectious mononucleosis and was admitted for treatment. She was later transferred to our hospital with elevated transaminases and a rapidly worsening clinical condition. Diagnostic workup revealed coagulation abnormalities, positive antiphospholipid and antinuclear antibodies, anti-dsDNA antibodies, and hypocomplementemia, all indicative of SLE. Given her young age and the aggressive nature of the disease, and considering the contraindication of renal biopsy due to bleeding risk, we opted for whole exome sequencing to identify potential pathogenic *genetic variants for targeted treatment options*.

SLE is characterised by tissue inflammation mediated by the activation of autoreactive lymphocytes and the subsequent production of autoantibodies, culminating in immune complex deposition and ultimately contributing to tissue and organ damage. Over 80 polygenic lupus susceptibility loci have been identified; targeted screening for these can enhance patient risk stratification and treatment optimisation (5, 6). Recent research strongly supports the involvement of various gene mutations as influential triggers for dysregulated or impaired responses in both the innate and adaptive immune systems, ultimately leading to a lupus-like syndrome (5). In SLE disease models, Fujimoto et al. (3) revealed that *SOCS1*-/- mice spontaneously develop a fatal disease due to aberrantly activated lymphocytes. The partial restoration of *SOCS1* in lymphoid cells rescues *SOCS1*-/- mice from early onset fatal disease. However, in these mutant mice with partial restoration of *SOCS1* expression, *SOCS1* levels were insufficient to effectively downregulate its target signaling. Consequently, these mice exhibited spontaneous hyperactivation of lymphocytes, elevated anti-DNA autoantibody levels, increased serum Ig, and glomerulonephritis with IgG deposition in the glomeruli. The observations in murine models led to the hypothesis that a deficiency in *SOCS1* accelerates the development of autoimmune phenotypes. Reduced *SOCS1* levels may impair the negative feedback on the JAK/STAT pathway, leading to unchecked cytokine production and a heightened risk of SLE development.

In 2020, Thaventhiran et al. (7) first characterised *SOCS1* haploinsufficiency in an index case, detailing recurrent bacterial infections, severe autoinflammation, and autoimmunity. Notably, Hadjadj et al. (2) subsequently reported two pediatric SLE patients (D1 and E1) with *SOCS1* insufficiency, who presented with early-onset disease (9–16 years old) and autosomal dominant inheritance, distinct from the adult female-predominant pattern of polygenic SLE. Both patients developed lupus nephritis with mesangial hypercellularity and C1q deposition, associated with impaired immune complex clearance and CD21^−^CD38^−^ B-cell expansion. Mechanistically, their lymphocytes showed hyperactive STAT1/STAT5 phosphorylation in response to IFN-γ and IL-2, mirroring the “cytokine hypersensitivity” observed in our patient's JAK/STAT pathway dysregulation.

Subsequently, a second case and her father, both with a similar clinical presentation of severe infections, atopy, and autoimmunity, were identified; the father also exhibited jaundice and unexplained liver disease. Lee et al. (8) reported on two additional patients with autoimmune cytopenias in the context of acute infections and one with paediatric multisystem inflammatory syndrome. They revealed heightened type I and II interferon responses, underscoring *SOCS1*'s pivotal role as a negative regulator of interferon pathways. Michniacki et al. (9) reported a pediatric patient with *SOCS1* insufficiency resulting from a complete gene deletion. Notably, the patient exhibited a remarkable therapeutic response to tofacitinib, a Janus kinase inhibitor known for its capacity to dampen interferon signaling pathways. Our patient's antiphospholipid antibody positivity and early liver involvement represent unique clinical features not explicitly reported in Hadjadj's cohort, highlighting the phenotypic variability of *SOCS1* insufficiency. Nevertheless, the shared themes of JAK-STAT hyperactivation and B-cell dysregulation across cases support *SOCS1* mutations as a monogenic driver of “cytokine-driven” SLE subsets. The response to glucocorticoids and mycophenolate mofetil in our patient, combined with the documented efficacy of JAK inhibitors in *SOCS1*-deficient models, reinforces the rationale for personalized therapy targeting this pathway. Type I interferonopathies are systemic disorders characterized by autoimmune and inflammatory traits. These conditions frequently manifest in early childhood, with more than 80% of cases commencing prior to the age of three (10). The heterogeneity of their clinical manifestations poses a significant challenge to early diagnosis. In the case of our patient, WES was instrumental in identifying the *SOCS1* frameshift variant, which was inherited from her mother. This genetic finding provided critical insights into the pathogenesis of the patient's SLE and paved the way for a more personalized treatment approach.

**Figure 2:**
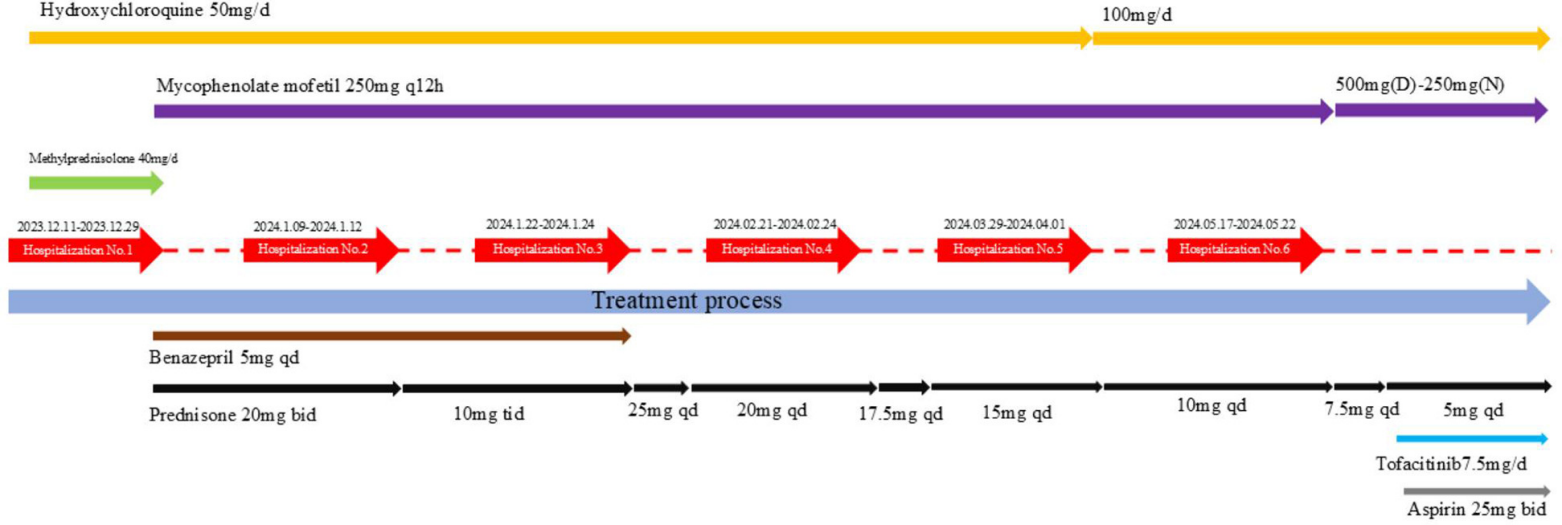
A detailed description of the treatment conditions.

During the initial six-month treatment period, glucocorticoids, hydroxychloroquine, and immunosuppressive agents formed the cornerstone of anti-inflammatory and immunosuppressive therapy for this SLE patient. The patient's condition gradually improved. Complement levels returned to normal, autoantibodies became negative, and proteinuria decreased. Fresh frozen plasma infusions kept her abnormal coagulation function under control. At present, the child is still under follow-up, with urine protein remaining negative. Her parents refused a renal pathological biopsy. However, due to the physical examination report indicating decreased serum complements C3 and C4, positive lupus anticoagulant, and persistently positive dsDNA, the parents consented to treatment with tofacitinib (weight >15–25 kg, 7.5 mg/day). A detailed description of the treatment conditions can be found in Figure 2. We chose tofacitinib over other JAK inhibitors for several reasons. First, the patient's clinical and laboratory findings indicated significant JAK-STAT pathway activation, and tofacitinib has shown efficacy in inhibiting this pathway in previous studies. Second, considering the patient's young age, tofacitinib was deemed a suitable option due to its relatively mild yet effective profile. Third, tofacitinib has been approved by the FDA for juvenile idiopathic arthritis, supporting its use in pediatric patients. Additionally, since its introduction in Chinese hospitals, tofacitinib has become more affordable and accessible, facilitating convenient access for our patient.

In conclusion, this case highlights the importance of genetic analysis, particularly in children with severe or atypical symptoms, a family history of relevant diseases, and individuals from consanguineous families. Incorporating Whole Exome Sequencing into the diagnostic workflow for paediatric-onset lupus cases enables a more accurate and timely aetiologic diagnosis, facilitating improved clinical management. Identifying pathogenic genes will aid in the discovery of novel therapeutic targets and the development of effective biomarkers.

## Data Availability

The original contributions presented in the study are included in the article/Supplementary Material, further inquiries can be directed to the corresponding author.
